# Editorial: Social determinants of psychological illness and well-being across the life course

**DOI:** 10.3389/fpsyg.2023.1215136

**Published:** 2023-06-07

**Authors:** Jingyi Wang, Ning Zhang, Ruimin Ma, Fei Yan

**Affiliations:** ^1^School of Public Health, NHC Key Laboratory of Health Technology Assessment, Fudan University, Shanghai, China; ^2^School of Public Health and The Second Affiliated Hospital, Zhejiang University School of Medicine, Hangzhou, China; ^3^Department of Psychological Medicine, Institute of Psychiatry, Psychology and Neuroscience (IoPPN), King's College London, London, United Kingdom

**Keywords:** social determinants, psychological illness, psychological well-being, mental health, life course

A good psychological state is integral to a person's health, high life satisfaction and overall functioning. There is increasing evidence that social, economic, environmental and political factors shape psychological illness and wellbeing across different stages of the life course (Alegria et al., 2018). There is a considerable need to understand social determinants of health at policy, community, organizational, interpersonal and intrapersonal levels, and employ a multi-layered and multi-sectoral approach to prevent psychological illness and to promote wellbeing (Rose-Clarke et al., 2020). It is also of significance that coordinated action needs to be taken to improve population psychological wellbeing from before birth, during early childhood, older childhood and adolescence, during family building and working ages, and at older ages. Taking a life-course perspective can demonstrate how risk factors at different stages of life influence psychological wellbeing or predispose people to psychological illness at a later stage (World Health Organization and Calouste Gulbenkian Foundation, 2014).

In our Research Topic, we include several contributions to various social determinants of common psychological illnesses across different stages of the life course. They provide empirical findings covering a wide range of domains, such as loneliness, social support, income, urban-rural household registration, childhood family risks, COVID-19 experiences, and strategies to deal with social determinants of psychological health.

First, Wang J. et al. provide an original research article of mental health problems among school-aged children after school reopening during the COVID-19 pandemic in eastern China. The authors examined the prevalence of emotional and behavioral problems in primary school students, and investigated the associations between psychological stressors, daily activities, social interactions, and mental health problems. They found that psychological stressors caused by school reopening and social distancing measures, and longer time involved in homework and computer games were associated with higher risk of emotional and behavioral problems, whereas higher level of engagement in physical exercise and more frequent social interactions with friends or relatives were related to lower risk.

The study by Wang C. et al. highlights the importance of giving and receiving acts of kindness with strangers. The authors used a mixed-methods single-group design to evaluate a participatory public mental health project. They reported that sending and receiving a card with goodwill messages may improve wellbeing, loneliness, sense of belonging and hope. Sending a kindness card enhanced personal fulfillment, leading to improvement in wellbeing. Receiving a kindness card enhanced self-esteem and established a sense of connection with others, which could reduce loneliness.

The contribution by Heidinger and Richter investigated the impact of COVID-19 experiences on mental health in older adults from 28 European countries, considering COVID-19 illness severity, and proximity to the infected individual. The authors provided empirical evidence that psychological burden was affected by severity of illness, with severe COVID-19 experiences in oneself or close networks as the strongest factor. Importantly, the research identified that even a less severe illness in the distant network (e.g., relatives outside household, neighbor/friend/colleague, caregiver or other) also had an unfavorable effect, emphasizing the necessity of population-wide pandemic control to safeguard people's mental health during COVID-19.

The study by Xinzhu examined the association between childhood family risks (CFRs) and mental health in Chinese older adults using a nationally representative sample, and investigated the moderating effect of age. The author reported that CFRs (low socio-economic status and broken family structure) were negatively associated with mental health in older participants, and identified the cumulative effect of CFRs on mental health. Additionally, the study noted that age moderated the association and the effect was attenuated as people age.

Yu et al. focus on urban-rural household registration, subjective social status and loneliness. They investigated the underlying mechanism of the discrepancies in anxiety and depressive symptoms between urban and rural university students in China. They found that the urban-rural household registration had both direct and indirect effect on emotional problems through subjective social status and loneliness. Preliminary evidence presented in their study suggests that reducing the disparity of social status and addressing loneliness are essential to improve mental health of university students.

The work by Li et al. examined the nonlinear relationship between income and mental health using a large-scale nationally representative sample in China. They found a U-shaped impact of individual income on depression level. At the lower income level, depressive symptoms declined as income increased. However, beyond middle income, further increases in income were associated with higher level of emotional problems. The authors discussed the clinical implications of their findings.

In the systematic review by Gómez-Zúñiga et al., the authors outline definitions of loneliness in the context of disability and relevant intervention strategies. Although consistency has not been reached in terms of single or multiple dimensions of loneliness, several intervention strategies have been developed to tackle loneliness among disabled people.

Finally, consistent with the need to improve social support and psychological health, Shin and Park elucidated how social support from various sources was differently related to psychological wellbeing among younger and older adults. They reported that social support from spouses and friends had the strongest associations with happiness and depressive symptoms, and the associations were mediated by the extent to which their needs for autonomy and relatedness were met.

As editors, we are delighted to introduce the eight articles to the readers of Frontiers in Psychology and the broader academic community on social determinants of mental health from the life course perspective. The studies in this Research Topic will have a positive impact on research in this field and have real-world implications for developing effective interventions to improve mental health and wellbeing for people at different stages of the life course. We hope that these contributions will stimulate new ideas and more rigorous research on social determinants of psychological illness and wellbeing across the life course.

## Author contributions

JW, NZ, RM, and FY contributed equally to the development of the outline of this editorial. JW wrote the first draft, which was revised and edited by NZ, RM, and FY. All authors approved the final version of the manuscript.
